# Evaluating policies and regulations used to control corruption among accounting officers in the public sector of South Africa: a systematic literature review

**DOI:** 10.3389/fsoc.2024.1371287

**Published:** 2024-10-24

**Authors:** Hester Vorster, Lilian Nwosu

**Affiliations:** GIFT, North-West University, Mahikeng, South Africa

**Keywords:** accounting officer, corruption, evaluation, accountability, public sector, implementation, planning

## Abstract

Corruption, a global phenomenon, affects countries worldwide, transcending geographic and political boundaries, and continues to escalate, partly due to the inefficiency of financial and legal institutions and lack of enforcement. This has significant implications for Africa, hindering democracy, development, and poverty alleviation efforts. Defined as the intentional misuse of official authority for personal benefit, corruption is often fueled by public sector financial managers or accounting officers embezzling funds. The increasing pressure on these officers to justify public fund usage, coupled with expectations for effective service delivery, may contribute to corrupt practices. This article presents a systematic literature review focused on evaluating policies and regulations to control corruption among accounting officers in South Africa’s public sector. The public sector in South Africa has recently seen a rise in corruption cases, with numerous incidents involving corrupt government officers, including accounting officers, gaining media attention. This situation prompted the need for the study, which systematically reviewed existing literature to identify gaps and weaknesses in current policies and regulations. The findings revealed non-compliance with anti- corruption policies and regulations, along with a lack of accountability among accounting officers. Additionally, the study observed that punishment measures for those implicated in misusing public funds are inconsistently implemented, highlighting a significant gap between policy and practice. These findings are critical for government policymakers, as they reveal areas needing improvement in anti-corruption policies and regulations. Issues identified include unqualified officers, poor delegation, embezzlement, and non-compliance. The study recommends that the government consistently apply strict measures against officials guilty of corruption, holding them accountable for their actions. It also advocates for the implementation of effective monitoring and evaluation systems to enforce anti-corruption policies and bridge the policy-practice gap. Emphasizing that good planning is as crucial as the implementation structures themselves, the study suggests that effective functioning requires both elements to work in tandem.

## Introduction

Corruption has become one of the world’s most serious issues. It is intrinsically inefficient, squandering resources, slowing *per capita* income growth, and lowering job possibilities (Dang, 2016). According to Rose (2018), corruption undermines trust in public authorities, resulting in decreased tax collections and poorer public service delivery. Corruption is defined as the theft of public resources by individuals in positions of power to serve the people (Krsteski, 2017). It can occur anywhere and is classified as either grand or petty based on the amount of money involved and the sector affected (Basabose and Basabose, 2019).

In South Africa, the public sector is tasked with using public funds to improve citizens’ lives through service delivery, not for profit-making (Moolman, 2021). Yet, according to Moolman (2021), many instances exist where public officers, including accounting officers, chief accounting officers, and managers, divert these funds for personal gain. The Head of the Special Investigation Unit in South Africa reported to Parliament in 2011 that between ZAR 25 millio n and ZAR 30 million from the government procurement budget were misappropriated (Krsteski, 2017). Furthermore, ZAR 215 million meant for public services was diverted to improve Nkandla, President Jacob Zuma’s private mansion at the time (Public Protector Report on Nkandla, 2014).

According to Dixit (2018), anti-corruption laws such as the Municipal Finance Management Act 56 of 2003 (MFMA), Treasury Regulations (National Treasury of South Africa, 2012), and Preventing and Combating Corruption Act 12 of 2004 (PCCA) are not properly applied (Rose, 2018). The OECD has expressed concern over South Africa’s low enforcement of legal frameworks (National Treasury, 2000). Separately, Pozsgai-Alvarez (2020) identifies a lack of accountability for maladministration and corruption in government finance as a major cause of theft. According to Krsteski (2017), corruption is an abuse of a public position that involves direct or indirect personal benefits and violations of policies, regulations, and ethics. Consequently, this study evaluates policies and regulations controlling corruption among accounting officers in the public sector in South Africa. Dang (2016) notes that corruption includes economic improprieties such as embezzlement or accepting bribes, and nepotism in public office appointments.

South Africa has faced notable corruption scandals that highlight the systemic nature of the problem. Aside from the Nkandla case, Zuma is still being prosecuted for the arms deal, and the Commission on State Capture’s extensive findings revealed the involvement of high- ranking officials and private entities, including the Gupta brothers and Atomstroyexport (Commission of Inquiry into Allegations of State Capture, 2024). The scandal concerning President Cyril Ramaphosa and the Phala Phala farm scam has highlighted the continuous corruption at the highest levels of government (Commission of Inquiry into Allegations of State Capture, 2024). These instances demonstrate the complicated and deeply established nature of corruption at the highest levels of government.

Beyond these high-profile cases, the function of accounting officers in such a corrupt environment raises serious concerns about their authority and responsibility (Odeku, 2019). Odeku (2019) further states that while accounting officers are responsible for managing public funds, their independence is frequently undermined by political meddling and senior officials’ influence. Given the practice of cadre deployment (Swanepoel, 2022), in which politically loyal persons are recruited to key posts, it is critical to consider whether accountability should be primarily with accounting officers or if ministers, who may select or dismiss them, and should carry more responsibility (Fagbadebo, 2019).

One challenging aspect is the role and agency of accounting officers in such an environment (Jeppesen, 2019). While accounting officers are responsible for managing public funds, their effectiveness can be compromised by political interference and the influence of senior officials (Botlhoko, 2017). The issue of cadre deployment, where politically loyal individuals are placed in key positions, raises questions about the independence and accountability of accounting officers (Zulu et al., 2022). According to Zulu et al. (2022), if they can be removed by ministers or pressured to act against ethical standards, their capacity to combat corruption is significantly undermined.

Furthermore, South Africa’s standing on the global stage, within the Southern African Development Community (SADC) region and as part of the BRICS group of emerging economies, reflects its ongoing struggles with corruption (Mlambo, 2017). International indices and rankings often highlight South Africa’s challenges in maintaining transparency and accountability in governance (Transparency International, 2023). Researching and publishing in this field also pose significant challenges.

Furthermore, as a member of the BRICS group of developing economies, South Africa’s corruption difficulties (Freddy et al., 2019) are particularly alarming, especially given the expectations of BRICS members to set an example in economic development and governance. Although South Africa is ranked higher on the Corruption Perceptions Index (CPI) than some of its BRICS rivals, corruption remains a major issue (Domashova and Politova, 2021). It has an impact on both internal administration and South Africa’s position in the BRICS (Freddy et al., 2019). While the OECD has taken a more proactive approach to promoting anti-corruption laws and prosecutions (Pasculli and Ryder, 2020; Findley et al., 2020), the prevalence of corruption in BRICS nations, especially South Africa, highlights the need for ongoing vigilance and change (Freddy et al., 2019).

Researchers risk potential threats to their personal safety and professional ramifications, which can hinder further investigation of the topic. This reality may contribute to a lower volume of information on corruption in South Africa than might be expected. Furthermore, conducting interviews or surveys with people who may be implicated in corrupt activities, such as current and retired accounting officials or high-profile characters, presents significant methodological hurdles. Even if access were allowed, the accuracy of the information obtained would be questioned. Furthermore, such research findings may be difficult to replicate in less democratic regimes, hampering efforts to fully understand and eliminate corruption.

Given this context, this study does not delve into the types of corruption in which accounting officers engage but rather evaluate the current policies and regulations controlling corruption among them in the South African public sector. This focus is critical, as the findings could serve as a roadmap for improving policy enforcement and developing strategies to strengthen existing policies and regulations against corruption in the South African context.

## Background and rationale for the study

Corruption is a pervasive issue impacting nearly every region of the world and is often regarded as one of the most critical global challenges (Suleiman and Othman, 2016). Many countries perceive it as alarmingly high, and it continues to escalate (Fagbemi and Olatunde, 2019; Pasculli and Ryder, 2019). Reports indicate that this increase is influenced by the low efficiency of financial and legal institutions, as well as their lack of enforcement (Tonoyan et al., 2010). Consequently, corruption continues to harm Africa by hampering democracy, development, and the ability to lift people out of poverty (Naidoo, 2012).

In South Africa, an overwhelming number of corruption scandals, especially in the public sector, are reported almost daily (Suleiman and Othman, 2016). During the 2019 State of the Province address (North West State of the Province Address, 2019), the premier of the Northwest Province highlighted progress on the status of corruption in the region. A notable example of these scandals is the Bosasa case, where accounting officers in the correctional services departments were implicated in deep-rooted corrupt activities (February, 2019; Styan and Vecchiatto, 2019). This clearly shows that corruption is a worldwide issue, underscoring the urgent need to ensure the effective and efficient implementation of policies and regulations to control all forms of corruption.

Given this context, it was deemed imperative by the researcher to conduct a study evaluating the current policies and regulations used to control corruption among accounting officers, as the number of such cases keeps rising (Sambo, 2017). Accounting officers must report corrupt activities according to the Department of National Treasury (National Treasury of South Africa, 2012) contracts management framework. However, recent reports indicate a growing number of corrupt accounting officers who have been suspended for failing to report other corrupt public officers, thus showing a lack of integrity and commitment (National Treasury, 2012). This unfortunate trend suggests that accounting officers are not only failing to comply with their duty to report corruption as mandated by the National Treasury, but they are also becoming perpetrators themselves. Munzhedzi (2016) believes that while there are policies and regulations in place to prevent accounting officers from engaging in corruption, these policies and regulations need to be strengthened.

## Problem statement and questions

Economic development is crucial for every country (Schumpeter and Swedberg, 2021); however, the public sector in South Africa has recently faced an increasing number of corruption cases (Shava and Mazenda, 2021), posing a risk to the country’s economy. This risk is further exacerbated by persistently high unemployment rates and the very poor economic growth achieved since 1994, which together hinder the country’s ability to achieve sustainable development (Jeke and Wanjuu, 2021). Dassah (2018a) reports that the top 10 corruption scandals in South Africa, including those in local government and parastatals, occurred in the public sector. This situation prompted the researcher to investigate potential gaps in the public sector. Additionally, in the North-West province, there has been a significant increase in the number of accounting officers who have been suspended and are under investigation, leading to the initiation of this study (Sethunyane, 2021). Furthermore, according to the National Prosecuting Authority (NPA) the number of government officials convicted of corruption increased by 38.4%, year on year (NPA, 2022).

Corruption in public sectors has been a dominant topic in the media and public discourse. For instance, state-owned enterprises such as South African Airways, the Passenger Rail Agency of South Africa (PRASA), and Eskom have made headlines due to mismanagement scandals (Dassah, 2018b). Dassah also states that South Africa has lost R700 billion to corruption since 1995, and in 2013, it was reported to lose R25 billion annually due to corrupt government procurement practices.

Corruption and unethical behavior continue to pose serious concerns for business growth and the South African economy (Manyaka and Nkuna, 2014). Sethunyane (2021) notes that corruption remains the biggest threat to democracy and to any potential social and economic reforms aimed at empowering the poor majority. While numerous studies have investigated the types of policies and regulations used to control corruption, little research has been done on evaluating the policies and regulations controlling corruption among accounting officers (Bruce, 2019; Šumah, 2018). Therefore, this study aims to bridge this gap by evaluating the current policies and regulations controlling corruption among accountants in public sectors. It also seeks to identify ways accounting officers can comply with these policies and regulations and the code of professional conduct.

The main research objective is to systematically review existing literature on policies and regulations controlling corruption among accounting officers in the public sector to identify gaps or opportunities. To achieve this main research objective, the Systematic Literature Review (SLR) approach will address the following objectives:

- To investigate the current policies and regulations used to control corruption among accounting officers in the public sector.
- To ascertain the roles of accounting officers in ensuring adherence to the current policies and regulations and code of professional conduct.
- To investigate the challenges in the current policies and regulations controlling corruption among accounting officers in the organization.
- To suggest possible interventions that can strengthen the existing policies and regulations controlling corruption among accounting officers in the public sector.

## Literature review

Literature indicates that corruption significantly impacts the livelihoods of ordinary South Africans and the broader economy (Masuku, 2019; Gossel, 2017; Gumede, 2019). A primary challenge in currentpolicies and regulations designed to control corruption is misinterpretation. For instance, Swart and Swanepoel (2019) highlight that a key issue in the interpretation of policies and regulations for controlling corruption among accounting officers in the public sector is non-compliance with Treasury Regulations, the Public Finance Management Act, and the Fraud and Corruption Act.

Furthermore, Oageng (2021) reported considerable misconduct among accounting officers in public sectors. The report cites the current Head of Department (HOD) in the Northwest Health Department, who is facing a disciplinary hearing for serious financial irregularities. Additionally, five other officials in the Department of Health face disciplinary charges related to fraud, corruption, and violations of Treasury policies and regulations, and the Public Finance Management Act (PFMA), involving over ZAR 350 million (Masuku, 2019). In another case, the Mahikeng Local Municipality suspended an accounting officer over allegations of investments made in contravention of adopted council policies and municipal investment regulations relating to VBS Mutual Bank. The officer’s conduct was also deemed a violation of policies and regulations due to a lack of due care (Gumede, 2019). However, Dassah (2018b) asserts that the government is actively working to eradicate corruption by appointing commissions.

## Contribution of the study

The existing literature on the evaluation of policies and regulations used to control corruption among accounting officers in public sectors has yet to fully embrace a SLR approach. This research offers several significant contributions. Firstly, by evaluating the current policies and regulations aimed at controlling corruption, the study provides new insights and potential strategies for accounting officers to mitigate corruption. Secondly, it might inform the government, particularly policymakers, through its findings and recommendations, highlighting areas in need of improvement in anti-corruption policies and regulations.

Furthermore, this study could assist the government in assessing whether the policies and regulations intended to reduce corruption in South Africa have been effectively implemented. It could also aid in adjusting these measures to ensure the equitable distribution of resources and achieve efficient service delivery, which is a primary mandate of government departments.

## Theoretical framework

The fraud triangle framework forms the basis for this study’s approach to addressing the research challenge. Originally introduced by Cressey in 1953 (Manurung and Hadian, 2013), the fraud triangle concept is widely employed to understand and manage fraud within organizations. The theory suggests that three essential elements—pressure, opportunity, and rationalization—must all be present for fraud to occur.

Pressure often drives individuals to engage in activities stemming from difficulties, personal challenges, or unrealistic work demands within the organization. The opportunity arises when a person may commit fraud without being detected. This usually happens in environments with lax internal controls, limited oversight, or ineffectual restrictions. Justifying behavior is what rationalization entails. People may believe their actions are valid, whether it is because they feel unfairly treated, think they are not adequately compensated, or plan to return the stolen money in the future.

**Figure 1:**
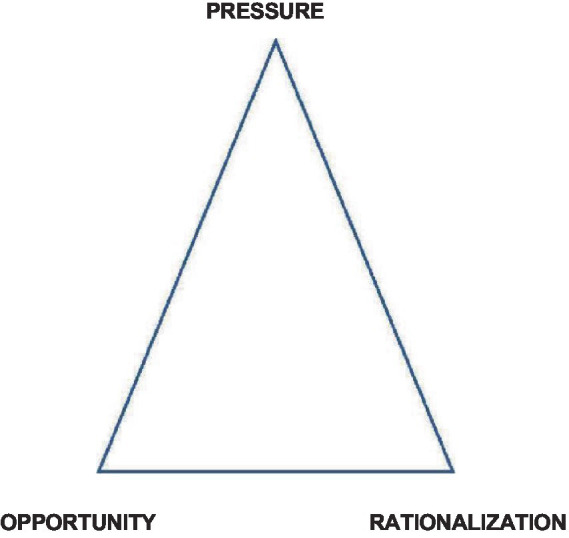
Fraud triangle by Cressey, 1953 (Manurung and Hadian, 2013).

Figure 1 below illustrates the components of the fraud triangle as conceptualized by Cressey (Manurung and Hadian, 2013).

### Application in analysis

The fraud triangle theory was systematically applied in the analysis by identifying and categorizing instances of pressure, opportunity, and rationalization among accounting officers.

#### Pressure

The study identified pressure by examining factors such as financial difficulties, personal challenges, and unrealistic performance expectations faced by accounting officers. For example, increasing demands on these officers to justify public fund usage and meet service delivery goals were found to contribute to corrupt practices.

#### Opportunities

Opportunities for corruption were identified by examining deficiencies in internal controls, a lack of oversight, and ineffective rules. According to the report, ineffective anti- corruption policies and regulations and uneven disciplinary actions presented an opportunity for accounting officials to commit fraud.

#### Rationalization

The study explored how accounting officers justified their corrupt actions, finding that rationalization often stemmed from perceived unfair treatment, inadequate compensation, or the belief that they would later reimburse the stolen funds. These officers often viewed their actions as necessary responses to systemic challenges.

### Research approach

This study utilized a SLR methodology. This approach enabled the researcher to comprehensively search for relevant literature, such as articles, government reports, and dissertations, across various search engines, thereby assisting in achieving the study’s objectives. A SLR is a method of literature review that systematically collects and critically analyzes multiple research studies or papers through a structured process. Essentially, a SLR identifies, selects, and critically appraises research to answer a specifically formulated question (Dewey and Drahota, 2016). In this process, the researchers classified existing literature into similar conceptual groups, systematically ordering and describing the results to evaluate their quality and relevance.

The primary aim of conducting a SLR on existing studies is to create a comprehensive overview, gather evidence on specific questions, and provide a summary of the existing literature on a given problem (Shahrol et al., 2020). A SLR was deemed appropriate for this study as it establishes a solid foundation for knowledge development and highlights areas where further research is needed. The SLR process in this study consisted of three main steps: the search process, inclusion and exclusion criteria, and quality evaluation. Each of these steps are briefly described below:

### Search process

This study adopted the literature search process using keywords and online journal articles databases, Google Scholar, Science Direct, Springer Link, Ebsco host, Sabinet, and IEEE **Xplore** Digital Library. The following keywords were searched as derived from the study objectives:

- The current policies and regulations are used to control corruption among accounting officers in the public sector.
- Roles of accounting officers in ensuring adherence to the current policies and regulations and code of professional conduct.
- The challenges with the current policies and regulations used to control corruption among accounting officers in the organization.
- Possible interventions that can strengthen policies and regulations that are used to control corruption among accounting officers in the public sectors

The use of these research objectives assisted the researchers in narrowing down the scope of the search and focusing on only related literature.

### Inclusion and exclusion criteria

Implementing inclusion and exclusion criteria is a crucial step in conducting a SLR. For this study, priority was given to papers published in peer-reviewed journals and conference proceedings. Searches will include documents published between 2000 and 2024. The inclusion of older documents is necessary to capture relevant policies and regulations that is still influential. An additional inclusion criterion was that the studies must be written in English and contain keywords relevant to the current policies and regulations used to control corruption among accounting officers in the public sector. Conversely, any paper that did not meet these specified criteria was automatically excluded from the SLR.

### Quality evaluation

To ensure the quality of the selected papers, the researcher established several guidelines to facilitate the selection of only the most relevant papers for this study. This step in the SLR process was crucial to guarantee that the chosen articles were relevant, valid, reliable, and directly related to the study’s objectives. For an article to be included in the evaluation process, it must provide a discussion of at least one current policy and regulation used to control corruption among accounting officers in the public sector. Additionally, it should offer an analysis of the strengths and weaknesses pertinent to the study’s main research objective (see Table 1).

**Table 1:** Related literature on the current policies and regulations used to control corruption among accounting officers in the public sectors.

Themes	Authors	Key findings
Current regulations used to control corruption among accounting officers in your organization	Dlomo (2017), (Republic of South Africa, PFMA, 1999), Oageng (2021), Magani and Gichure (2018)	Public Finance Management Act (PFMA) (Act 1 of 1999).
	Maclean (2012), Selby (2018)	Municipal Finance Management Act (MFMA) (Act 56 of 2003).
	Swart and Swanepoel (2019), Dassah (2018a,b)	Constitution of the Republic of South Africa (Act 108 of 1996)
	Masegare and Ngoepe (2018), PWC (2016)	King Code IV
Roles of accounting officers in ensuring adherence to the current regulations and code of professional conduct	Ramos (2001), National Treasury (2000)	In-year management, monitoring and reporting.
	Marais and Odendaal (2008)	Establish effective internal controls.
	National Treasury (2012)	Improve expenditure management and transfers
	Selby (2018)	Responsible for budgetary processes.
	National Treasury (2000)	Responsible for Banking arrangements
	National Treasury (2000)	Complete financial statements on time
	Madue (2007)	Delegation of responsibilities
	RSA (1999), National Treasury, 2012	Responsibility for regarding unauthorized, irregular and wasteful expenditure
Challenges/weaknesses/loopholes in the current regulations used to control corruption among accounting officers in the organisation	Government Gazette (2007), Laubscher (2012) Mfuru et al., (2018) Edoun (2015), Naidoo (2012)	Unqualified accounting officers
	Sibanda (2017), Oageng (2021)	Inappropriate delegation of authority
	Dzomira (2017), Mazibuko and Fourie (2017)	Embezzlement of public funds and corruption
	Moloto and Lethoko (2018), AGSA (2019), AGSA (2020) Theletsane and Fourie (2014)	Lack of Implementation on policies and regulations aimed at controlling corruption
	Shabalala (2013), MFMA (2003)	Poor budgeting and cash mismanagement
	Xhati (2015), Swart and Swanepoel (2019)	Non-compliance of policies and regulations
Way(s) to strengthen the current regulations used to control corruption among accounting officers in the organization	Munzhedzi (2016), Kanyane and Sausi (2015), Fombad (2015), National Treasury (2000)	Accountability
	Khanyile (2016), National Treasury, 2012	Consistent disciplinary measures/punishments
	Laubscher (2012), Motubatse (2016), Jacobs (2019), Seitheisho (2017)	Skills and technical expertise
	Mfuru et al. (2018), National Treasury (2000), National Treasury, 2012	Effective planning and preparation procedures

## Discussion of themes

This section provides a detailed discussion of the current policies and regulations used to control corruption among accounting officers in South African government departments.

### Public Finance Management Act (PFMA) (Act 1 of 1999)

Dlomo (2017) states that the PFMA, Act No. 1 of 1999, was enacted by the South African Parliament to ensure effective and improved financial practices in government. The Act grants significant powers to accounting officers/authorities, enabling them to manage their financial affairs within the guidelines of prescribed norms and standards. The primary objective of the PFMA, as outlined in the Act (1999), is to ensure transparency, accountability, and sound management of revenue, expenditure, assets, and liabilities of the institutions it governs. Oageng (2021) emphasizes that the PFMA was aimed at reducing over-expenditure in provinces and public entities, including national departments, and was designed to enhance expenditure management by requiring periodic financial reporting to the central government.

Magani and Gichure (2018) note that public finance management reform focuses on transparency, governance enhancement, sensible allocation of financial resources, and accountability. Swart and Swanepoel (2019) report that the South African government is currently experiencing high levels of corruption and irregularities. The PFMA governs the public sector by regulating financial management, ensuring efficient and effective management of revenues, expenditures, assets, and liabilities, and stipulating the responsibilities of those entrusted with financial management.

Thutshini (2016) explains that the purpose of the PFMA is to assist authorities and accounting officers of compliant establishments in maintaining adherence to all relevant compliance requirements. Maclean (2012) adds that the PFMA outlines the responsibilities of accounting officers and mandates each government department to develop and implement a Fraud Prevention Plan. Oageng (2021) views the PFMA as a valuable tool for accounting officers managing government assets.

The Act specifies that accounting officers are responsible for managing resources and are accountable for their utilization. It establishes clear lines of accountability and broad frameworks of best practices that managers can adopt or adapt, as necessary. However, Dassah (2018a) clarifies that the Act does not intend to make accounting officers overly cautious to the point of failing to deliver agreed outputs in their departmental budgets for fear of contravening the PFMA. Accounting officers who underspend or underperform, which must be regularly monitored by the executive authority, are also at risk of violating the Act and facing the corresponding sanctions.

### Municipal Finance Management Act (MFMA) (Act 56 of 2003)

The Local Government Municipal Finance Management Act 56 of 2003 aims to prevent waste and ensure efficient and transparent governance, with a focus on ensuring that municipal resources effectively reach the poor (Maclean, 2012). Selby (2018) simplifies this by stating that the MFMA’s objective is to ensure sound and sustainable management of the financial affairs of municipalities and to establish treasury norms and standards within the local sphere of government. The Act also provides a framework for municipalities regarding borrowing and outlines conditions for both short- and long-term loans.

Additionally, the MFMA addresses financial challenges in local government, detailing the responsibilities of accounting officers and municipal councils (Selby, 2018). It places accounting officers at the forefront of the municipal budgetary process. The accounting officer, often the municipal manager, is tasked with implementing the municipal budget in line with Section 54(d). This section mandates that the mayor instructs the accounting officer to execute the budget according to the service delivery and budget implementation plan.

Moreover, the accounting officer plays a vital role in advising political office-bearers on administrative matters and in preventing unauthorized, irregular, and wasteful expenditures. As Maclean (2012) notes, it is the duty of the accounting officer to promptly inform the mayor, the MEC for Local Government in the province, and the Auditor-General in writing about any potential risks of incurring such expenditure.

### Constitution of the Republic of South Africa (Act 108 of 1996)

The Constitution of the Republic of South Africa, Act 108 of 1996, establishes in Chapter 1, Section 2, that the Constitution is the supreme law of the Republic. Any law or conduct inconsistent with it is invalid, and the obligations it imposes must be fulfilled. Consequently, it can be inferred that all acts related to financial management must align with the Constitution.

Financial officials, Supply Chain Management (SCM) officials, Chief Financial Officers (CFOs), Accounting Officers (AOs), and other key players in the public sector are mandated to abide by and uphold the Constitution (Swart and Swanepoel, 2019). These officials are expected to be accountable and to uphold constitutional principles. Chapter 3, Section 40(1) of the Constitution, enacted in RSA in 1996, established three spheres of government: National, Provincial, and Local governments. These spheres were created to serve the people of South Africa.

Chapter 13, Section 217(1) of the Constitution mandates that procurement be conducted through a supply chain management system that is fair, equitable, transparent, competitive, and cost- effective (Dassah, 2018a). Furthermore, according to the Constitution, accounting officers are responsible for managing the resources within their purview while being accountable for their use. Chapter 10, Section 195(1) emphasizes that all three spheres of government should operate effectively and efficiently in resource usage, ensuring economic viability and accountability (Swart and Swanepoel, 2019).

### King code IV

In South Africa, the corporate governance framework applicable to both the private and public sectors is known as the King IV report (Masegare and Ngoepe, 2018). Distinct from previous reports, the King IV report extends its application to state departments, including national, provincial, and local government administrations covered under the PFMA or MFMA, as well as to public institutions or functionaries operating under the Constitution or other legislation (Mgijima, 2018).

The King IV report emphasizes that “risk, as a cornerstone of governance and risk governance, is very different from the mere implementation of risk management” (PWC, 2016). According to them, it challenges the leadership structures of organizations to ensure they are effectively managing risk. In line with this, both the PFMA and the MFMA explicitly assign the responsibility of risk management to the accounting officer, who is the administrative head of the organization. In the context of municipalities, this role typically falls to the municipal manager. Essentially, the King IV report places the onus on accounting officers in national departments to proactively identify and manage key risk areas.

## Roles of accounting officers in ensuring adherence to the current policies and regulations and code of professional conduct

### In-year management, monitoring, and reporting

According to Ramos (2001:16), the PFMA mandates accounting officers to immediately act as managers, ensuring effective mechanisms for the in-year management of resources. They are also responsible for monitoring progress on the department’s operational plan, including the budget, and for acting on monthly and quarterly reports. These reports are then submitted to the executive authority and the treasury.

Through performance evaluation, accounting officers, executive authorities, and other stakeholders can determine whether their departments are achieving the objectives or outcomes outlined in their strategic plans (National Treasury, 2000). It is, therefore, crucial for accounting officers to ensure that staff involved in these processes have the necessary capacity and can interpret and, where required, act on the information produced.

### Establish effective internal controls

Internal controls refer to the systems (manual or electronic), procedures, and processes implemented to minimize risks, including financial consequences, which a department may face due to fraud, negligence, error, incapacity, or other causes (Ramos, 2001:16). In compliance with the PFMA, accounting officers are expected to evaluate the appropriateness of existing controls, assess the risks facing the department, and introduce necessary changes to the system of internal control. However, Marais and Odendaal (2008) found that internal auditors and accounting officers still require effective training in handling fraud and related irregular activities.

### Improve expenditure management and transfers

Ramos (2001) emphasizes the importance of proper planning by accounting officers before spending or transferring funds or benefits. Any transfer not specified in an appropriation act is considered unauthorized expenditure. Transfers to entities within the same sphere of government or to a private institution must be reported as per the PFMA and its policies and regulations. Transfers from a national department to a province or municipality should align with schedules in the Division of Revenue Act (DoRA) (Act No. 24 of 2024). and be reported accordingly.

Similarly, transfers from a provincial department to a municipality must be reported under section 13 of the DoRA. A transfer from a national department to a municipality, routed through a province, constitutes a direct charge on that province’s revenue fund. Ramos (2001) also notes that fiscal dumping (transferring funds late in the financial year to hide underspending by a national department) may amount to financial misconduct. Furthermore, the PFMA mandates that, unless contracted otherwise, payments should be made within 30 days of receiving an invoice. Delays in payment by accounting officers could undermine government objectives, such as supporting small, medium, and microenterprises (National Treasury, 2012).

### The role of an accounting officer regarding budgetary processes

Selby (2018) explains that the accounting officer, also known as the municipal manager, is responsible for implementing the municipal budget after the mayor has completed all steps outlined in Section 54(d). This section mandates the mayor to instruct the accounting officer to ensure that the budget is implemented in accordance with the Service Delivery and Budget Implementation Plan. Additionally, Section 3 of the Local Government Municipal Finance Management Act 56 of 2003 highlights the importance of public consultation, stating that any revisions to the Service Delivery and Budget Implementation Plan must be made public promptly by the mayor.

Selby (2018) also notes that Section 22 of the MFMA emphasizes the publication of the annual budget. This section requires the accounting officer to make the annual budget public and invite the local community to submit representations in connection with the budget. It is crucial for the accounting officer to submit the annual budget in both printed and electronic formats to the National Treasury and the relevant provincial treasury.

### Banking arrangements

The PFMA stipulates that accounting officers must take full responsibility for ensuring that all revenue received by their department is deposited only in the relevant revenue fund, with any exceptions being in accordance with Section 13(1) of the PFMA. Consequently, accounting officers must ensure that all suspense accounts are cleared and correctly allocated to the relevant cost centers each month. Any uncleared items should be promptly reported to the CFO. Failure to clear suspense accounts monthly could expose the accounting officer to charges of financial misconduct (National Treasury, 2000).

### Complete financial statements on time

A key role of accounting officers, in adherence to current policies and regulations and the code of professional conduct, is to submit annual financial statements to the Auditor-General within two months of the fiscal year-end. Achieving this timeframe can be challenging unless routine month-end procedures are systematically completed throughout the year. Therefore, the accounting officer must ensure that the necessary measures are in place for producing high-quality reports (National Treasury, 2000). It is important to note that the Auditor-General will not complete poorly prepared financial statements submitted by departments. In such instances, not only can these substandard financial statements be referred to the department, but they may also lead to charges of financial misconduct. Consequently, the accounting officer must ensure that the systems and financial staff within the department can prepare high-quality financial statements within the stipulated two-month period. The quality and timeliness of these reports are critical, as the Auditor-General will comment on both aspects.

### Delegation of responsibilities

The PFMA, Act 1 of 1999, mandates new delegations of financial responsibilities to appropriate officials within departments. This is essential for effective departmental management and for distributing management responsibilities among senior managers, as it is impractical for the accounting officer to personally undertake all the tasks required by the department. The Act permits the accounting officer to delegate any power or duty under the Act in writing to an individual official. For instance, Madue (2007) notes that accounting officers are expected to appoint CFOs as part of their senior management to help fulfill certain responsibilities. However, such delegation does not absolve the accounting officer of responsibility for exercising the delegated power or duty. The delegator must ensure that systems and processes are adequate to document, monitor, and review the exercise of these powers or assigned duties. In essence, all officials in the department are accountable for their specific areas of responsibility and should adhere to their official duties and those delegated to them to prevent unnecessary conflict.

### Responsibility regarding unauthorized, irregular, and wasteful expenditure

Sections 38(1)(c)(ii) and 51(1)(b)(ii) of the PFMA require accounting officers to take appropriate steps to prevent fruitless and wasteful, irregular, or unauthorized expenditure (RSA, 1999). In cases where such expenditure occurs, they are authorized to report it immediately in writing to the relevant treasury (RSA, 1999: section 38(1)(g)). Additionally, accounting officers are responsible for reporting on material losses incurred due to such expenditure in the annual report and financial statements of the national department (RSA, 1999: section 40(3)(b)(i)). To support the accounting officer in these duties, paragraph 14.18 of the Treasury Regulations (National Treasury, 2012) mandate that any department employee who discovers unauthorized, irregular, or fruitless and wasteful expenditure must report it immediately to the accounting officer.

Selby (2018) also emphasizes that one of the accounting officer’s responsibilities is to provide guidance to political office bearers on administrative issues. The accounting officer plays a vital role in preventing unauthorized, irregular, and wasteful expenditures. However, the occurrence of irregular, unauthorized, wasteful, and fruitless expenditures has been steadily increasing since 2016 in South Africa, affecting various government departments (AGSA, 2019). Section 32(4) of the MFMA outlines the responsibility of the accounting officer in advising a council about the likelihood of incurring such expenditure. The accounting officer must promptly inform the mayor, the MEC for Local Government in the province, and the Auditor-General, in writing, of:

- Any unauthorized, irregular, or fruitless and wasteful expenditure incurred by the municipalities.
- Whether any person is responsible for or under investigation for such expenditure?
- The steps that have been taken.

As mandated by various policies and laws, accounting officers are expected to make significant progress in these areas. Their role is to ensure adherence to current policies and regulations and the code of professional conduct, which should be included in their departmental plans. As financial managers, it is crucial that they perform their duties as detailed in these legislations to ensure equal and fair distribution of resources to the community through effective service delivery.

## Challenges/weaknesses/loopholes in the current policies and regulations used to control corruption among accounting officers in the organization

### Unqualified accounting officers

Chapter 2 (1) (2) (3) of the National Treasury in the Government Gazette No. 29658 (Government Gazette, 2007) stipulates that the accounting officer of a municipality must possess the skills, experience, and capacity necessary to assume and fulfill the responsibilities and exercise the functions and powers assigned by the Act. Consequently, the National Treasury (2012) issued a circular concerning the competency of officials who occupy executive positions within the local sphere of government to ensure that municipalities can achieve their objectives (Selby, 2018:49–50). However, Oageng (2021) suggests that the onset of financial negligence in the public sector is linked to a lack of skills required to manage public accounts. Laubscher (2012) also points out that financial management remains a challenge in the government, often due to appointing unqualified individuals, such as accounting officers.

Political interests also appear to play a significant role in the lack of skilled personnel in some municipal departments. Mfuru et al. (2018) note that political affiliation has influenced the appointment of public officials in higher positions. Edoun (2015:10) observed that “public enterprises are the safe haven par excellence through which political leaders redistribute wealth by giving employment to comrades who lack the necessary skills and knowledge to manage.”

This lack of skills, particularly in accounting among politicians, leads to increased fraud and corruption, resulting in unclean audit findings (Sibanda, 2017; Dintwe and Masiloane, 2014; Naidoo, 2012). Oageng (2021) asserts that it is inappropriate for state funds to be managed by politicians. Thus, employing qualified personnel capable of managing government funds effectively and efficiently is paramount, and the influence of political leaders pursuing personal interests at the expense of community needs should be minimized.

### Inappropriate delegation of authority

As previously mentioned, the PFMA allows for the proper delegation of authority. However, Oageng (2021) notes instances of power abuse for personal gain, often neglecting the needs of poor citizens by public officials in higher authority. Sibanda (2017) highlights that the shifting of management responsibilities to lower levels while retaining overall control is a trait of maladministration within the government. Despite administrative systems, guidelines, policies, and regulations in place, they are often disregarded. In such cases, the accounting officer should establish a system of delegation of authority to enhance operational efficiency and provide adequate checks and balances (Sibanda, 2017).

### Embezzlement of public funds and corruption

The absence of effective financial management and the continuous incurrence of unnecessary expenditure remain challenges hindering service delivery (Dzomira, 2017). Mazibuko and Fourie (2017) concur, noting that the government continues to face challenges in controlling contract execution and evaluation. This lack of oversight creates avenues for escalating corruption. Sibanda (2017) explains that corruption often leads to the misuse of public funds, where these funds are either misappropriated by select individuals or entities or diverted from government revenue into private hands.

### Lack of implementation of policies and regulations aimed at controlling corruption

Proper corruption policies and regulation implementation requires adequate capacity in the form of structures staffed with fully skilled and professional personnel. However, the shortage of qualified and skilled personnel in South Africa is a recurring challenge, affecting not only the government but also other sectors (Moloto and Lethoko, 2018). This scarcity of skilled workforce continues to be a key constraint for proper municipal financial management and Supply Chain Management (SCM) implementation.

Key officials such as Accounting Officers, CFOs, and Senior Managers must possess the necessary skills and experience to fulfill their responsibilities and exercise their powers (AGSA, 2019, AGSA, 2020). These officials play a crucial leadership role in their institutions. Theletsane and Fourie (2014) assert that the failure of municipalities to appoint competent accounting officers can hinder public financial management. Matlala (2018) emphasizes that sound financial governance is critical, and without proper personnel management, municipalities will continue facing financial management challenges.

In a study by Nyide (2022) on municipal financial management practices for improved compliance with SCM regulations, most participants indicated that enforcing policies aimed at stopping corruption remains challenging. Some participants believed that while some policies are sound, their implementation is poor. A significant number also agreed that policies and laws are implemented, but municipalities struggle to fully implement them due _15_ to senior management instability and political interference.

Khosa (2003) found that policy implementation has suffered from the absence of a people- driven process, highlighting a lack of managerial expertise as a key gap between policy and implementation. He also pointed out that the interpretation of policies and directives remains challenging due to the absence of standard criteria and a lack of effective integration of strategic planning and performance management in legislative compliance. Fourie (2007) contends that although the PFMA is well-written, its implementation and enforcement are unsatisfactory.

From the above discussion, it can be inferred that there is a lack of effective implementation of legislation aimed at combating corruption in South Africa, resulting in a substantial gap between policy and practice. This necessitates strict repercussions for those responsible for implementing these policies and laws. The government should also ensure consistent evaluation or monitoring processes where submission of reports on implementation progress is mandatory and enforce hefty punishments for non-adherence.

### Poor budgeting and cash mismanagement

Effective budgeting is crucial in any organization, as it can significantly influence the success or failure of the entity. In government departments, poor budgeting can lead to subpar service delivery, whereas effective budgeting contributes to departmental success. Shabalala (2013:20) refers to Section 85 (1) (a) and (e) of the PFMA, which addresses the reporting of financial embezzlement in the public sector. The PFMA aims to modernize budgetary management within the public sector and reduce fraud, corruption, and waste.

Under the Act, accounting officers are responsible for overseeing budgetary management and adhering to sanctions against budgetary misconduct. However, Oageng (2021) found that accounting officers often set budgets lower than necessary, leading to poor service delivery. Oageng (2021:57) quotes a study participant: “Future allocations are mostly guided by the current performance, therefore, noncompliance with PFMA leads to under-collection of revenue and thus budget cuts in future years. When such happens, the budgets put in place might be tempered to make up the lapses.”

The MFMA Act (56 of 2003) states that cash management is a tool to control spending, implement the budget efficiently, minimize the cost of municipal borrowings, and maximize the opportunity cost of resources (MFMA, 2003). Hence, transformation in budgeting is essential to minimize cases of poor service delivery.

### Non-compliance with policies and regulations

The PFMA outlines those public offices must use financial resources in accordance with its guidelines. Xhati (2015) asserts that all officials within a department must comply with the PFMA to provide effective services. Managers with the requisite knowledge are well-placed to implement the PFMA. Yet, challenges persist in government departments. Swart and Swanepoel (2019) observe that noncompliance with PFMA requirements negatively affects South Africa’s development potential.

Noncompliance with PFMA guidelines leads to the misallocation of funds across all spheres, negatively impacting the economy and reducing funds for crucial areas (Oageng, 2021). Moreover, noncompliance with budgetary and expenditure guidelines contravenes the PFMA (2000) (AGSA, 2019). The effects of noncompliance extend across departments and society, resulting in insufficient resources for good governance.

The implications can be severe, affecting the government’s functionality and impacting available resources. Swart and Swanepoel (2019) revealed that noncompliance with PFMA requirements by the Northwest Provincial departments and public entities led to billions of rands not reaching intended beneficiaries. This noncompliance results in the government’s inability to adjust spending levels appropriately, negatively affecting GDP and service delivery.

Oageng (2021) notes that despite efforts by accounting officers, noncompliance with the PFMA remains a challenge. Nyide (2022) mentions that, although the PFMA is enforced by financial management directors, gaps still hinder departmental success. Essentially, noncompliance with the PFMA has serious repercussions for the economic growth and development of the country.

WAY(S) TO STRENGTHEN THE CURRENT POLICIES AND REGULATIONS USED TO CONTROL CORRUPTION AMONG ACCOUNTING OFFICERS IN THE ORGANISATION.

### Accountability

A significant challenge in current policies and regulations aimed at controlling corruption is the lack of accountability. In South Africa, the concept of accountability originates from the Constitution of the (Republic of South Africa, PFMA, 1999). Munzhedzi (2016) notes that in the South African public service, procurement through the tender process has been used to address historic discriminatory policies and regulations, benefiting the previously disadvantaged population. The (Republic of South Africa, PFMA, 1999) (Act 1 of 1999) aims to ensure accountability and effective governance of the income, expenses, assets, and liabilities of public sector institutions (Kanyane and Sausi, 2015). The Act establishes clear lines of accountability and provides broad frameworks of best practices for managers to adopt or adapt, as necessary. However, Khanyile (2016) points out that local government faces challenges, including poor financial accountability.

Magani and Gichure (2018) affirm that governance problems in the public sector, entwined with animosity and corruption, tarnish the entire system, necessitating competent management and accountability. It is essential to hold heads of departments and other officials accountable for their performance, emphasizing the efficient, effective, economical, and transparent use of departmental resources, which may be more critical than traditional compliance-based accountability. The National Treasury (2000) acknowledges that shifting towards this form of accountability will take several years.

The government must establish practical accountability structures and implement consistent laws to penalize those liable for wrongdoing. The National Treasury (2000) states that the performance contract of the accounting officer should be based on the agreed plan. Fombad (2015) underscores that South Africa requires political leadership and multiple measures to enhance accountability to improve service delivery and fulfil national and international development initiatives.

### Consistent disciplinary measures/punishments

Disciplinary measures specified in various laws should be consistently followed in corruption cases. Although these measures are clearly defined in the Acts, the extent to which corruption cases are reported and disciplinary actions are taken against perpetrators is often unclear. For instance, accounting officers are expected to ensure that incompetent or dishonest departmental officials are subjected to prescribed disciplinary steps. If the accounting officer fails in this duty, it becomes the responsibility of the executive authority to act against the accounting officer (National Treasury, 2012). A lack of consequences for misbehavior or misconduct has been identified as a contributing factor to the failure of local government. It is crucial for the relevant officers to execute their duties effectively and report any corruption occurrences so that appropriate corrective steps can be taken. The principle that actions speak louder than words apply; the laws against corruption must be enforced to deter deceitful acts such as embezzlement of public funds and corruption.

### Skills and technical expertise

Laubscher (2012) notes that municipalities have undergone significant changes, necessitating high levels of expertise among key players. However, there is a noted shortage of capacity and technical expertise in local municipalities for managing financial responsibilities (Moloto and Lethoko, 2018; Jacobs, 2019). Seitheisho (2019) views the scarcity of expertise in local government as a major factor in the lack of provision of basic services to communities.

Swanepoel (2022) points out that many municipalities are characterized by a lack of proper expertise, skills, and capacity, leading to non-compliance with SCM policy and regulations and preventing the sector from achieving clean audits. This has resulted in an overreliance on consultants for basic financial management activities (Ambe, 2016). Motubatse (2016) adds that the weak link between financial management and clean audit outcomes can be attributed to a general lack of competencies in the public service and specific shortages of qualified personnel in accounting sections. Nyide (2022) identifies a lack of capacity as a root cause of weak service delivery, particularly in financial management units.

Seitheisho (2019) asserts that the African continent, including its local governments, suffers from inadequate capacity and technical expertise to handle financial responsibilities. It is, therefore, imperative for the government to employ qualified personnel who are experts in their fields and to consider training less competent employees through regular workshops.

The AGSA Report on Education and Health (AGSA, 2011) details that due to a lack of technical and project management skills, contracts are not effectively monitored, leading to delays, and reduced cost-effectiveness. The report also found that procurement officials’ inability to maintain proper records undermines accountability, as documents cannot be submitted for auditing. Thus, it is vital for the government to employ skilled personnel in various capacities and provide ongoing training through workshops to ensure they are up to date with evolving practices.

### Effective planning and preparation procedures

Effective planning is crucial for the successful implementation and operation of any organization. Mfuru et al. (2018) state that planning is the first of the management functions, followed by organizing, leading, and controlling. The National Treasury (2000) emphasizes that accounting officers must ensure that the essential components for effective planning and preparation procedures are in place. These preparations include employing qualified and experienced CFOs and accountants, establishing effective internal controls, constituting an audit committee, and meeting in-year management requirements.

The National Treasury (2012) reported that accounting officers are responsible for achieving the operational plan by fostering commitment to the plan across the department and more importantly, by ensuring that all managers accept their financial management responsibilities.

From this discussion, it is evident that accounting officers who fully embrace their responsibilities for effective planning and preparation are less likely to encounter challenges in their roles. Therefore, accounting officers need to exceed the call of duty in executing their roles and responsibilities as outlined in the policies and regulations aimed at addressing and combating corruption in the governmental departments where they serve.

## Findings and discussion

The findings found noncompliance with anti-corruption laws and procedures, as well as a lack of responsibility among accounting officers. Furthermore, the study found that disciplinary mechanisms for those involved in squandering public funds are inconsistently enforced, indicating a considerable gap between policy and practice. These findings are essential for government authorities because they identify opportunities for improvement in anti-corruption legislation and regulations.

### Challenges in current policies and regulations

Several issues have been discovered with the current regulatory structure, including the nomination of unqualified accounting officers, incorrect delegation of responsibility, misappropriation of public funds, policy implementation failures, inadequate budgeting, and regulatory noncompliance. These obstacles weaken the effectiveness of anti-corruption measures and contribute to the continuance of corrupt behaviors among accounting officers.

### Application in analysis

The fraud triangle theory was used systematically to analyze the data by detecting and categorizing instances of pressure, opportunity, and rationalization among accounting officers: Accounting officers may experience pressure due to financial issues, personal concerns, or unrealistic performance expectations. For example, increased pressure on accounting officers to justify public fund expenditure and achieve service delivery goals has been linked to unethical activities.

Opportunities for corruption were found through assessments of internal control flaws, supervision gaps, and ineffective policies. The study found that a lack of effective anti- corruption policies and regulations and inconsistent penalty mechanisms increased opportunities for fraud among accounting officers.

#### Rationalization

The study explored accounting officers’ justifications for corrupt behavior. This included justifying their actions based on perceived unjust treatment, insufficient remuneration, or the expectation that they would refund the stolen monies later. The study discovered that accounting officers frequently justified their actions by perceiving them as required to deal with systemic difficulties.

#### Guided recommendations

The fraud triangle theory influenced several significant recommendations for increasing anti- corruption measures:

Addressing Pressure: To relieve pressure, the report recommends providing accounting officers with enough assistance and resources, setting reasonable performance objectives, and increasing job security to insulate them from political influence.
Reducing Opportunities: To lessen the likelihood of fraud, the report suggests enhancing internal controls and oversight procedures. This includes putting in place comprehensive monitoring and assessment procedures to ensure compliance with anti- corruption rules and close the policy-practice gap.
Challenging Rationalization: To prevent the rationalization of corrupt behavior, the study advises consistent disciplinary sanctions and vigorous implementation of anti-corruption laws.
Ensuring that all authorities are held accountable for their conduct is critical to discouraging the justification of corrupt behavior.

## Conclusion and recommendations

South Africa possesses well-documented policies and regulations aimed at combating corruption within governmental departments, particularly those guiding the roles of accounting officers. These policies and regulations include explicit measures against officials, such as accounting officers, who fail to comply, emphasizing accountability. However, the extent to which these corrective measures are implemented is unsatisfactory, leading to a high rate of corruption cases in the South African government. This study infers a significant gap between policy and practice.

To address this issue, the study recommends that the government needs stricter enforcement of the current measures. Additionally, the issue may be a lack of political will or the selective prosecution of officials while protecting politicians. The government should impose stringent penalties on officials found guilty of wrongdoing as a form of corrective action.

The study further suggests the establishment of effective monitoring and evaluation systems for the implementation of anti-corruption policies and regulations. Adequate reporting to the necessary authorities is crucial and must be taken seriously; therefore, there should be frequent monitoring of budgets and expenditures. It is also essential that accounting officers are assured of job security. Their role in reporting officials who misuse government resources is critical, and they may face threats from those in higher positions wielding political influence. Thus, a clause protecting accounting officers from harm is necessary to enable them to perform their duties effectively and confidently.

Implementing these recommendations will alleviate the burden on accounting officers, who are not only the official managers responsible for the equal distribution of resources but also the financial managers of government resources. They are accountable for any failure in executing such roles, and thus, ensuring their protection and support is imperative for the successful management of government resources and the reduction of corruption.

### Future research

While the current study has made major contributions to understanding corruption, particularly in the context of South Africa, there are several areas that require more investigation. Future studies could benefit from a more in-depth look at the function of whistleblowing and related policies in addressing corruption. Furthermore, because the landscape of corruption is constantly changing, subsequent studies should include more recent data and articles from the 2020s to keep the research current and relevant. These efforts could yield significant information and lead to more effective anti-corruption strategies in the future.
